# Genome-Wide Organization of GATA1 and TAL1 Determined at High Resolution

**DOI:** 10.1128/MCB.00806-15

**Published:** 2015-12-18

**Authors:** G. Celine Han, Vinesh Vinayachandran, Alain R. Bataille, Bongsoo Park, Ka Yim Chan-Salis, Cheryl A. Keller, Maria Long, Shaun Mahony, Ross C. Hardison, B. Franklin Pugh

**Affiliations:** Center for Eukaryotic Gene Regulation, Center for Comparative Genomics and Bioinformatics, Department of Biochemistry and Molecular Biology, The Pennsylvania State University, University Park, Pennsylvania, USA

## Abstract

Erythroid development and differentiation from multiprogenitor cells into red blood cells requires precise transcriptional regulation. Key erythroid transcription factors, GATA1 and TAL1, cooperate, along with other proteins, to regulate many aspects of this process. How GATA1 and TAL1 are juxtaposed along the DNA and their cognate DNA binding site across the mouse genome remains unclear. We applied high-resolution ChIP-exo (chromatin immunoprecipitation followed by 5′-to-3′ exonuclease treatment and then massively parallel DNA sequencing) to GATA1 and TAL1 to study their positional organization across the mouse genome during GATA1-dependent maturation. Two complementary methods, MultiGPS and peak pairing, were used to determine high-confidence binding locations by ChIP-exo. We identified ∼10,000 GATA1 and ∼15,000 TAL1 locations, which were essentially confirmed by ChIP-seq (chromatin immunoprecipitation followed by massively parallel DNA sequencing). Of these, ∼4,000 locations were bound by both GATA1 and TAL1. About three-quarters of them were tightly linked to a partial E-box located 7 or 8 bp upstream of a WGATAA motif. Both TAL1 and GATA1 generated distinct characteristic ChIP-exo peaks around WGATAA motifs that reflect their positional arrangement within a complex. We show that TAL1 and GATA1 form a precisely organized complex at a compound motif consisting of a TG 7 or 8 bp upstream of a WGATAA motif across thousands of genomic locations.

## INTRODUCTION

Hematopoietic stem cells undergo self-renewal and differentiation into many blood cell lineages: erythroid cells (red blood cells), lymphocytes, and myelocytes (including megakaryocytes) (1). Differentiation into red blood cells, referred to as erythropoiesis, requires several transcription factors, such as GATA1, TAL1 (also referred to as SCL), LMO2, LDB1, FOG1, and KLF1 (2–4). GATA factors are essential for hematopoiesis, as shown by the anemic phenotypes of mouse knockout mutations and the leukemias and lymphomas associated with mutations of the human genes (5). GATA1 is a master regulator of differentiation, proliferation, and apoptosis of red blood cells and megakaryocytic cells (6). Mutations in or misregulation of GATA1 leads to transient myeloproliferative disorder and acute megakaryoblastic leukemia in infants with Down syndrome (6). TAL1, a basic helix-loop-helix hematopoietic transcription factor, is required for multiple functions in hematopoiesis, including terminal differentiation of red blood cells (7). TAL1 makes an obligatory heterodimer with E2A basic helix-loop-helix proteins, such as E47, and along with other proteins listed above, nucleates a complex that includes GATA1 bound to its cognate site. An important missing component in our understanding of how GATA1, TAL1, and their cognate DNA recognition motif function is their precise local organization across the genome.

Several ChIP-seq (chromatin immunoprecipitation followed by massively parallel DNA sequencing) studies have been performed on GATA1 (8–11) and TAL1 (7, 11, 12) in erythroid cell lines and primary cells as a major step toward understanding the genome-wide binding properties of GATA1 and TAL1 and, ultimately, the biology of GATA1 and TAL1 regulation. ChIP-seq robustly identifies the genomic locations of these proteins and therefore the associated biology. However, the limited resolution of the assay places confidence limits on precise binding locations. Indeed, the broad binding regions of ChIP-seq, which span over 100 bp, make it challenging to decipher the relevant motif when numerous motifs may be present within an occupied segment; they also may not allow any differences in binding patterns and distances between GATA1 and TAL1 binding locations at cooccupied locations to be distinguished.

Estimates of the number of binding locations vary considerably among ChIP-seq studies. Three studies identified 4,000 to 6,000 *in vivo* binding sites for GATA1 in mouse MEL erythroleukemia cells expressing a tagged form of GATA1 (10, 11) or human K562 erythroleukemia cells (9). Another study identified >15,000 sites occupied in mouse G1E-ER4 cells, where the GATA1 genes had been knocked out of mouse embryonic stem cells (G1E) and then restored under the artificial control of estradiol (8). Recently, ENCODE has reported ∼12,000 GATA1 and ∼2,000 to 8,000 TAL1 binding locations in the mouse G1E-ER4 cell system after 24 h of GATA1 activation and 24,000 to 60,000 sites bound by GATA1 in MEL cells (13–15). While some differences in the number of bound locations may result from occupancy level thresholding, other factors, such as cell type, antibody quality, peak-calling methods, and data quality, might also contribute to the differences. Nonetheless, the substantial number of high-confidence locations has provided substantial insight into the network of genes involved in erythroid development.

GATA1 recognizes the WGATAA (International Union of Pure and Applied Chemistry [IUPAC] consensus) motif, whereas TAL1 and its heterodimeric partner, E2A, recognize the E-box (CANNTG) in solution (16, 17). Complexes containing both GATA1 and TAL1 tend to have their cognate motifs spaced 9 bp apart (18, 19). However, the determinants of binding may be more complex than those captured by their individual consensus motifs. First, the highly conserved WGATAA consensus sites are insufficient to accurately predict *in vivo* GATA1 binding (20, 21). Second, TAL1/E2A complexes have been suggested to bind to DNA with GATA1 without the need for an E-box (22), although another study found CTG upstream of WGATAA (CTG[N_7–8_]WGATA), in peaks cooccupied by GATA1 and TAL1 (11). Third, the *in vivo* developmental functions of TAL1 do not require its DNA binding region (23). Fourth, DNA site selection studies show that, in complex with other proteins, including GATA1, the TAL1/E2A complex prefers to bind GATA1/E-box composite motifs, rather than an E-box alone (18). Moreover, the GATA binding site motif is a stronger determinant of TAL1 occupancy than is the E-box (7, 18, 19, 24). How these erythroid transcription factors are positionally organized around their cognate motif remains unclear and is the focus of this study.

GATA1 activates or represses transcription, depending on the context with other transcription factors. GATA1-TAL1/E2A complexes induce gene expression, while GATA1 without TAL1 represses it (8, 11, 12, 25). However, GATA1 and KLF1 cooccupancy leads to gene activation and may be TAL1 independent. This was indicated by the low overlap between GATA1/KLF1 and GATA1/TAL1 regions ascertained in that study (26). How the interplay and cooccupancy of various proteins in GATA1 multiprotein complexes activate or repress transcription, and therefore regulate the erythroid differentiation program, is not well understood (19). As one step toward this goal, we focused on defining more precisely the structural interactions between GATA1, TAL1, and its DNA binding site on a genomic scale using the ChIP-exo (chromatin immunoprecipitation followed by 5′-to-3′ exonuclease treatment and then massively parallel DNA sequencing) assay (27). Since ChIP-exo is a high-precision derivative of ChIP-seq, we expect substantial overlap between locations identified by both methods. As such, the resulting gene-regulatory networks are expected to be the same. Therefore, this study is focused on the structural aspects and parameters of binding discernible by ChIP-exo but not by ChIP-seq rather than on gene-regulatory pathways.

## MATERIALS AND METHODS

### Cell culture.

Cells were cultured as described previously (28). G1E and G1E-ER4 cells were grown in Iscove's modified Dulbecco's medium (IMDM) with 15% fetal calf serum, 2 U/ml erythropoietin, and 50 ng/ml kit ligand. To activate the conditional GATA1-ER, cells were cultured in the presence of 10^−7^ mol/liter beta-estradiol for 24 h (29), and G1E-ER4 cells from 0 h, 3 h, and 24 h were obtained during this activation process. Then, sonicated chromatin materials of G1E and G1E-ER4 0-h, 3-h, and 24-h cells were prepared by standard methods.

### ChIP-exo.

With prepared sonicated chromatin, ChIP was performed on GATA1 (antibody sc265 L1609) and TAL1 (sc12984). Standard ChIP methods were used, followed by lambda exonuclease treatment and library construction, as described previously (30). Sequencing was performed using an AB 5500xl genetic analyzer, Illumina HiSeq2000, and Illumina NextSeq. For single-end reads from HiSeq, base calls were performed using CASAVA version 1.7 (Illumina), and for paired-end reads from NextSeq, base calls were performed using Bcl2fq version 2.15 (Illumina). ChIP-exo reads were aligned to the mm9 genome assembly using Bowtie 1.00 for SOLiD and BWA (version 0.6.2 for HiSeq single-end reads and version 0.7.9a for NextSeq paired-end reads) with default options. Non-uniquely mapped reads were filtered out in order to remove the reads with low mapping quality. The sequencing statistics are reported in Table S1 in the supplemental material.

### Determination of binding locations using intersection of peak pairing and MultiGPS (see Fig. 2).

Prior to peak pairing, tags from biological replicates were merged after demonstrating their reproducibility. During peak pairing, sequence read distributions were used to identify peaks using the strand-separate peak-calling algorithm in GeneTrack (parameters: sigma = 5, exclusion zone = 10) (31). After peaks within the blacklist regions were removed (32, 33), the peaks were paired if the plus-strand peak was within 5 bp upstream or 25 bp downstream of the minus-strand peak. Peak pairs that were enriched >2-fold over an input control with a *q* value of <0.05 were selected (*q* values are adjusted *P* values from binomial tests for multiple-hypothesis testing). To collect binding locations that are present at one or more time points (“union” of binding locations), binding locations within 40 bp were determined to be bound at multiple time points, while those more than 40 bp apart were determined to be time-point-specific binding.

MultiGPS is designed to detect binding locations across multiple conditions while characterizing differential binding between conditions (34). MultiGPS detected binding locations across multiple conditions with reads enriched >2-fold over the input control and *q* values of <0.05. The MultiGPS commands were as follows: o -geninfo mm9.info -threads 4 -*q* 0.05 -*d* reb1_chipexo.distrib.txt -exclude blacklist.bed -design design_gata1 -verbose -probshared 0.99 -poissongausspb -medianscale -prlogconf -5 -memepath usr/bin -mememinw 6 -mememaxw 16 -seq mm9. “-*q*” is for the minimum *Q* value (corrected *P* value) of reported binding events, and “-*d*” is for the binding event read distribution file for initializing models; the true distribution of reads around binding events is estimated during MultiGPS training (52). GATA1 and TAL1 binding locations are reported in Table S2 in the supplemental material.

### Homotypic clustering of transcription factor binding analysis (see Fig. 3).

Occurrences of the distance between adjacent binding location midpoints were calculated. Binding location midpoints that were closer than 500 bp from the adjacent binding locations were clustered as one binding unit, a homotypic cluster of transcription factor binding, while the median genomic coordinate was taken as the binding location. When a binding location was more than 500 bp from a nearby binding location, it was considered a noncluster. The closest transcription start site (TSS) of the mm9 RefSeq gene was assigned to the binding location as the target gene. Gene expression fold changes were obtained from transcriptome sequencing (RNA-seq) of G1E and G1E-ER4 cells across time points (14, 15).

### Binding around WGATAA motifs (see Fig. 4 and 5).

MEME (multiple EM for motif elicitation) analysis, which finds ungapped motifs in unaligned DNA, RNA, or protein sequences, was performed on 80-bp sequences surrounding the top 500 highly occupied binding locations of GATA1 and TAL1. Occurrences of top motifs were scanned across the binding locations using FIMO (find individual motif occurrences) (*P* < 10^−3^) to classify the binding locations by the presence of cognate motifs. FIMO analysis searches a sequence database for occurrences of known motifs. This program treats each motif independently and reports all putative motif occurrences below a specified *P* value threshold. The binding locations were centered on the most significant motifs if a motif was present within a 40-bp distance. When a motif was present multiple times, the most significant motif (with a more significant *P* value) was chosen. Figures displaying strand-specific sequencing tags of merged time points represent the merged raw data (G1E-ER4 cells at 0 h, 3 h, and 24 h for GATA1, and G1E and G1E-ER4 cells at 0 h, 3 h, and 24 h for TAL1) without normalization.

When sorting the binding locations by occupancy around the WGATAA motif, GATA1 occupancy was measured as tags within 25 bp upstream to 25 bp downstream, while TAL1 occupancy was calculated as tags within 40 bp upstream to 30 bp downstream.

When 7,927 GATA1 binding sites with a WGATAA motif were sorted by TAL1 occupancy, approximately one-third (2,997 locations) showed cooccupancy of GATA1 and TAL1, while the remainder (4,930 locations) showed only GATA1 occupancy. Then, the frequencies of dinucleotide TG distances from WGATAA were calculated for both GATA1- and TAL1-cooccupied sites and GATA1-only-bound sites. Among the top 2,997 GATA1- and TAL1-cooccupied WGATAA sites, TG was present at 1,650 sites between 7 and 9 bp distant from WGATAA. To examine the sequence composition of a full E-box (NNNNTG), the 4 upstream sequences of 1,650 TG sites were examined.

### TAL1-only binding locations around the E-box (see Fig. 4A).

Cooccupancy of GATA1 and TAL1 binding locations were determined if the binding location midpoints were within 40 bp. When TAL1-only binding locations with a WGATAA motif (FIMO *P* value < 10^−3^) were removed, the remaining sequences were mostly enriched with the E-box CAGMTG motif. Then, these binding locations were further classified by the presence or absence of an E-box motif within 40 bp of the binding location midpoint.

### ChIP-exo and ChIP-seq comparison (see Fig. 9).

Overlap between the binding locations of ChIP-exo and ChIP-seq (15) for the same factor in identical cell types was examined. Binding location midpoints lying within a 40-bp distance were defined as overlap. As a measure of consistency, Spearman correlation of log_10_-transformed read counts of ChIP-exo and ChIP-seq within a 400-bp window surrounding binding location midpoints of all ChIP-seq (*n* = 22,729) and ChIP-exo (*n* = 10,168) reads were calculated. A heat map scatter plot was drawn using the LSD package of R. Lastly, the distributions of GATA1 G1E-ER4 3-h cell ChIP-exo reads (total tag count normalized) around ChIP-seq-only-bound locations (*n* = 14,071) were plotted.

### Differential occupancy of GATA1 across time points (see Fig. 10).

EdgeR (Bioconductor [https://bioconductor.org/packages/release/bioc/html/edgeR.html]) was run internally, along with MultiGPS, to call differential binding events between time points. *k*-means clustering was performed on log_2_-fold change of GATA1 occupancy between G1E-ER4 cells at 3 h/0 h, 24 h/3 h, and 24 h/0 h. For analyses of occupancy levels, data were normalized so that the total tag counts were equal across all time points of a given factor and mapped around the binding location midpoints. Binding locations were assigned to the closest annotated RefSeq gene to calculate their gene expression fold change between time points (15) and their distances from the transcription start site.

### Microarray data accession number.

Sequencing data are available at the NCBI Sequence Read Archive under accession number GSE68964.

## RESULTS

In this study, we employed G1E (GATA1^−^) mouse erythroid cells (35, 36). Reintroduction of GATA1 fused to the estrogen binding domain provides a cell system (G1E-ER4) for synchronous and homogeneous erythroid maturation in response to estradiol treatment (28). Here, we examined these cells before and after 3 and 24 h of estradiol treatment.

### GATA1 and TAL1 locations determined by ChIP-exo.

**FIG 1:**
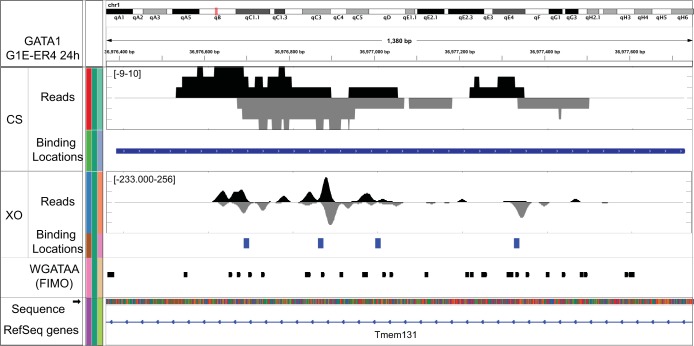
Determination of binding locations using intersection of MultiGPS and union of peak pairs. Shown is a browser shot of ChIP-exo tag 5′ ends for GATA1 measured by ChIP-seq (CS) and ChIP-exo (XO) on chromosome 1 from coordinates 36,976,370 to 36,977,750. Tag locations were smoothed (20-bp moving average). The tag density on the lower (negative) strand is shown as an inverted plot.The locations of WGATAA (*P* < 10^−3^) and RefSeq genes are shown below through the Integrative Genomics Viewer (IGV) browser.

TAL1 is abundantly expressed in G1E cells, and its production declines very little after activation of the G1E-ER4 cell line (data not shown). Compared to ChIP-seq, the use of exonucleases in ChIP-exo substantially reduces background, which often represents a substantial proportion of the ChIP-seq signal (27). Therefore, in addition to its higher precision, a practical advantage of ChIP-exo is that fewer total tags are required to achieve a similar depth of identification of locations (reported in Table S1 in the supplemental material) (27). In ChIP-exo, the 5′ ends of the sequencing tags correspond to a point about 6 bp upstream (5′) of a protein-DNA cross-link where movement of the lambda single-stranded exonuclease is blocked. For a single protein-DNA cross-link, a block occurs on both strands, resulting in two peaks of 5′ ends located on opposite strands and separated by about 12 bp in the 3′ direction. Pairing of these peaks provides a potential resolution of a few base pairs (27). In ChIP-seq, the 5′ ends of sequenced tags correspond to random breakpoints in sonicated solubilized chromatin and have less resolution than in ChIP-exo. Unlike ChIP-exo, ChIP-seq cannot resolve individual points of cross-linking within an individual binding location. Resolution is essential where proteins bind in closely clustered locations. For example, at many genomic locations, ChIP-seq detects GATA1 as a broad peak, whereas ChIP-exo reports many individual peaks, some of which correspond to separate WGATAA motifs (Fig. 1). Even when the signal track for ChIP-seq suggests multiple binding sites, peak-calling algorithms may combine them into a single peak.

**FIG 2:**
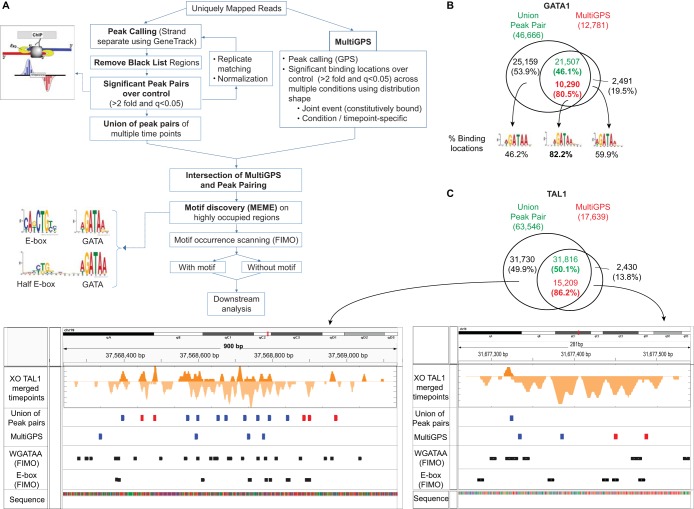
Peak-pairing and MultiGPS comparison of ChIP-exo location calling. (A) Workflow of ChIP-exo data analysis. Binding locations with high confidence were determined by obtaining the intersection of the union of significant peak pairs from multiple time points (left) and binding events determined by MultiGPS (right). Motif discovery analysis using MEME showed enrichment of WGATAA, E-box, and composite half E-box/WGATAA motifs on these stringent binding locations and validated both methods. To classify binding locations by the presence of motifs, FIMO and downstream analyses were conducted. (B) Venn diagrams showing overlap of GATA1 binding locations determined by the union of peak pairs and MultiGPS. MultiGPS binding locations positioned within a 100-bp window from the union of peak pairs were determined as final binding locations and percentages (*n* = 10,290). The most enriched MEME motifs and their occurrence frequencies from intersection and outersects of the two methods are shown below. (C) Binding locations detected by only one method, either peak pairing or MultiGPS. Binding locations called by the union of peak pairing and MultiGPS are shown in the Venn diagram. A browser shot of the 5′ end of TAL1 ChIP-exo tags surrounding the binding locations detected only by peak pairing is shown in the left browser diagram and that for MultiGPS in the right browser diagram. Binding locations at the intersection (blue) or outersection (red) of two methods are highlighted. WGATAA and E-box motifs are shown in black.

As is common with ChIP assays, the signal intensities at many locations may be relatively low, resulting in substantial numbers of false positives. False positives may be reduced by placing tighter positional constraints on relative ChIP-exo peak locations, but this potentially produces more false negatives. To identify potential alternative modes of binding, we allowed some positional flexibility. To balance these opposing stringencies, we implemented and compared two complementary approaches to identify binding locations: peak pairing and MultiGPS (34) (Fig. 2A). Peak pairing and MultiGPS are commonly used for ChIP-exo and ChIP-seq analysis, respectively, but have not previously been compared.

MultiGPS uses integrated machine learning to call binding events that are consistent with the learned binding pattern across multiple time points and biological replicates. MultiGPS allows the determination of binding events that are shared across conditions or are condition specific, using the binding pattern profile. Both methods required ChIP-exo signals to be at least 2-fold enriched over the input control, and a *q* value of <0.05 (*q* values are multiple-hypothesis-testing adjusted *P* values from binomial tests).

In an effort to examine comprehensive binding locations across all time points, we considered all statistically significant peak pairs arising from 0, 3, and 24 h of GATA1 activation. Peak pairing yielded ∼47,000 initial GATA1 candidate cross-linking points (which are not necessarily distinct binding locations) in mouse G1E-ER4 cells, whereas MultiGPS yielded ∼13,000 initial locations. Often, distinct peak pairs were very close together (<20 bp), and MultiGPS modeled them as a single location. Consequently, ∼10,000 (80%) MultiGPS locations contained ∼20,000 peak pairs (Fig. 2B; see Table S2 in the supplemental material). This intersect was highly enriched with the WGATAA motif (motif *P* value threshold = 10^−3^), thereby providing a general validation of the binding locations. Approximately 80% (7,927/10,290) contained the WGATAA motif. Thus, we detected and further analyzed ∼8,000 GATA1-bound WGATAA binding sites in differentiating mouse G1E-ER4 cells. These locations were detected by peak pairing and MultiGPS and contained a WGATAA motif and therefore represent a high-confidence set of locations. The remaining ∼2,000 sites, which were detected by both methods but lacked a WGATAA motif, may include some combination of noncognate DNA interactions, interactions with degenerate WGATAA motifs that fell below our bioinformatics detection threshold, and false positives.

When comparing the binding locations that were separately detected by MultiGPS and by peak pairing, the outersects, consisting of calls made by only one method, were less enriched with WGATAA motifs (60% and 46% versus 82%). Thus, while highly enriched with true positives, those called by only one method either have a higher false-positive rate or involve noncanonical cross-linking patterns that are not picked up by peak pairing. Representative examples of how calls at WGATAA sites can be made by only one method and not the other are shown in Fig. 2C (for TAL1). Whereas peak pairing could detect simple one-coordinate peaks in each pair, MultiGPS discounted them. In contrast, many binding locations that were detected only by MultiGPS were predominantly enriched with tags on only one strand or had sparsely distributed tags, thereby precluding peak pairing. Moreover, low-occupancy peak pairs often occurred at noncognate locations in the “shadows” of robust cognate binding events, which MultiGPS rolled into a single location. Also, tags may be piled up on both strands and are detected by peak pairing but not by MultiGPS, since they do not fit the distribution shape that MultiGPS deems to be a consensus. This analysis highlights the advantages and limits of both methods. Conservative estimates of locations might then use only the intersection resulting from the two methods but might have substantial numbers of false negatives, whereas more comprehensive estimates might include the union of the two methods but might have substantially more false positives.

When TAL1 was examined, the intersection of the two location-calling methods resulted in ∼15,000 candidate locations detected in one or more GATA1 activation time points (see Table S2 in the supplemental material). Therefore, MultiGPS peaks that overlapped with peak pairing were taken as higher-confidence binding locations, resulting in 10,290 GATA1 binding locations and 15,209 TAL1 binding locations. These are about 75% and 200%, respectively, of those reported previously by ChIP-seq.

**FIG 3:**
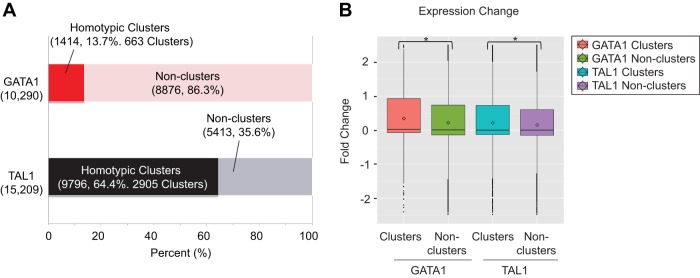
GATA1 and TAL1 locations reside in clusters. (A) Percentages of GATA1 and TAL1 binding locations in homotypic clusters among the total binding locations. A cluster is defined as adjacent binding locations that are <500 bp apart. In the case of GATA1, 13.7% (1,414) of the binding locations form 663 clusters, while 64.4% (9,796) of the TAL1 binding locations form 2,905 clusters. (B) Expression change of the closest gene to a homotypic cluster or noncluster for GATA1 and TAL1. Expression change was calculated as the log_2_-fold change of gene expression between G1E-ER4 cell induction for 24 h relative to that for 0 h. Significant differences (*P* < 0.05) using the Mann-Whitney test are indicated by asterisks. Boxes denote the first and third quartiles. Horizontal lines, circles, and vertical "whiskers" denote median, mean, and the limits of the data, respectively.

One advantage of ChIP-exo is its ability to detect two closely bound factors that would be called as the same binding location with ChIP-seq data. We therefore examined whether GATA1 ChIP-exo locations occurred in clusters. Among 10,290 GATA1 binding locations, 14% (1,414 GATA1 binding locations) were less than 500 bp from each other, forming 663 GATA1 clusters (Fig. 3A) with approximately two GATA1 locations per homotypic cluster. Surprisingly, a substantial portion (64% [9,796]) of TAL1 binding locations formed 2,905 TAL1 clusters (<500 bp) (Fig. 3A). On average, approximately three TAL1 locations formed a homotypic cluster. The nearest genes to a cluster showed greater average gene expression change upon GATA1 activation than nonhomotypic clusters for both GATA1 and TAL1 (Fig. 3B), suggesting that clustering, on average, results in higher levels of gene expression.

### A structural model for the genome-wide cobinding of TAL1 and GATA1.

**FIG 4:**
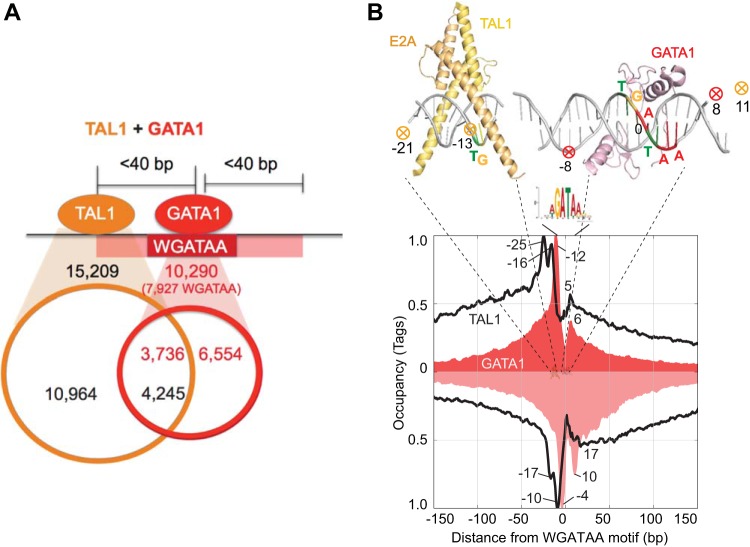
Genome-wide structural organization of GATA1/TAL1 complexes. (A) Venn diagram showing the overlap of TAL1 and/or GATA1 binding locations further classified as TAL1-only, GATA1/TAL1-cooccupied, and GATA1-only binding locations. Cooccupancy was defined as having midpoints <40 bp apart. (B) Composite distribution of TAL1 (black trace) and GATA1 (red-shaded trace) ChIP-exo tag 5′ ends around the WGATAA motif (the underlined A is set to zero) at GATA1 binding locations. Tags that mapped to the reverse strand that contained the WGATAA motif are displayed as inverted traces. The data were plotted as a moving average of 5 bp. Above the plot are X-ray structure images of TAL1 and GATA1 bound to cognate DNA sites (Protein Data Bank [PDB] accession codes 3VEK for GATA1 [J. M. Matthews, unpublished data] and 2YPB for TAL1 [SCL:E47] [17]). The circled X indicates the deduced site of cross-linking of GATA1 (red) and TAL1 (gold).

Though GATA1 and TAL1 have been known to work together, a high-resolution view of their precise positioning within a complex on DNA has not been examined *in vivo*, particularly on a genomic scale. Further, although cooccupancy by GATA1 and TAL1 is known to activate gene expression, the recognition motif for their cooccupancy still remains poorly identified. To this end, we compared the ChIP-exo binding locations of GATA1 and TAL1 (Fig. 4A) to determine the regions of cobinding and to analyze their underlying DNA sequence. Within a defined occupancy threshold, we identified 3,736 GATA1-bound GATA motif locations that also contained 4,245 TAL1 binding locations within 40 bp. The remaining 6,554 GATA1-bound GATA motif locations contained either no or low (i.e., subthreshold) levels of TAL1. Similarly, the remaining 10,964 TAL1 locations contained either no or subthreshold levels of GATA1. These “TAL1-only” locations were enriched with the same motif (described below) seen for GATA1/TAL1-cobound locations, indicating that a different GATA factor might be bound instead of GATA1. GATA1 is a member of a set of related GATA binding proteins.

In an effort to explore the genomic organization of GATA1 and TAL1 around the WGATAA motif, we plotted GATA1 ChIP-exo tag 5′ ends around all 7,927 WGATAA motifs (motif *P* value < 10^−3^) that were enriched in the 10,290 GATA1 binding locations (Fig. 4B, red-shaded plot). Remarkably, GATA1 displayed a double-peak-pair pattern around the WGATAA motif, with a peak pair located at each end of the motif. This is similar to what we have seen with many other proteins, including CTCF (27) and p53 (37), where cross-linking typically occurs at the edges of protein/DNA complexes. For GATA1, a major and a minor peak pair were observed, with the two pairs 16 bp apart. These points of cross-linking align precisely with structural models of the DNA binding domain of GATA1 in complex with DNA (shown in Fig. 4B, top), which indicates these models likely reflect the binding structure of GATA1 bound throughout the genome. Cross-linking to the left side of WGATAA was stronger than on the right side, which likely reflects differential reactivity between an appropriate cross-linkable amino acid and the DNA on the two sides. This level of structural congruity between genomic binding events and crystal structures has thus far been described in metazoans only for FoxA1 (38), the glucocorticoid receptor (39, 40), and the DMRT transcription factor (41) but to our knowledge represents the first genome-wide structural assessment of multiple components within a heteromeric complex.

TAL1 also displayed two major peak pairs, but the pairs were situated to the left of WGATAA sites (Fig. 4B, black trace). Their midpoints were 21 and 13 bp upstream (more 5′) of the WGATAA midpoint. The two inferred points of cross-linking were about 8 bp apart, which agrees with the modeled structure of TAL1/E47 with an E-box (18). A minor level of TAL1 cross-linking was observed on the right side of the WGATAA motif. This cross-linking did not match the double-peak pattern of GATA1 and thus largely rules out TAL1 cross-linking indirectly to DNA via cross-links to GATA1. Instead, we suspect that TAL1, in complex with GATA1 and other proteins, may also be in close proximity to DNA on the distal side of GATA1 (in addition to its main proximal-side interactions).

**FIG 5:**
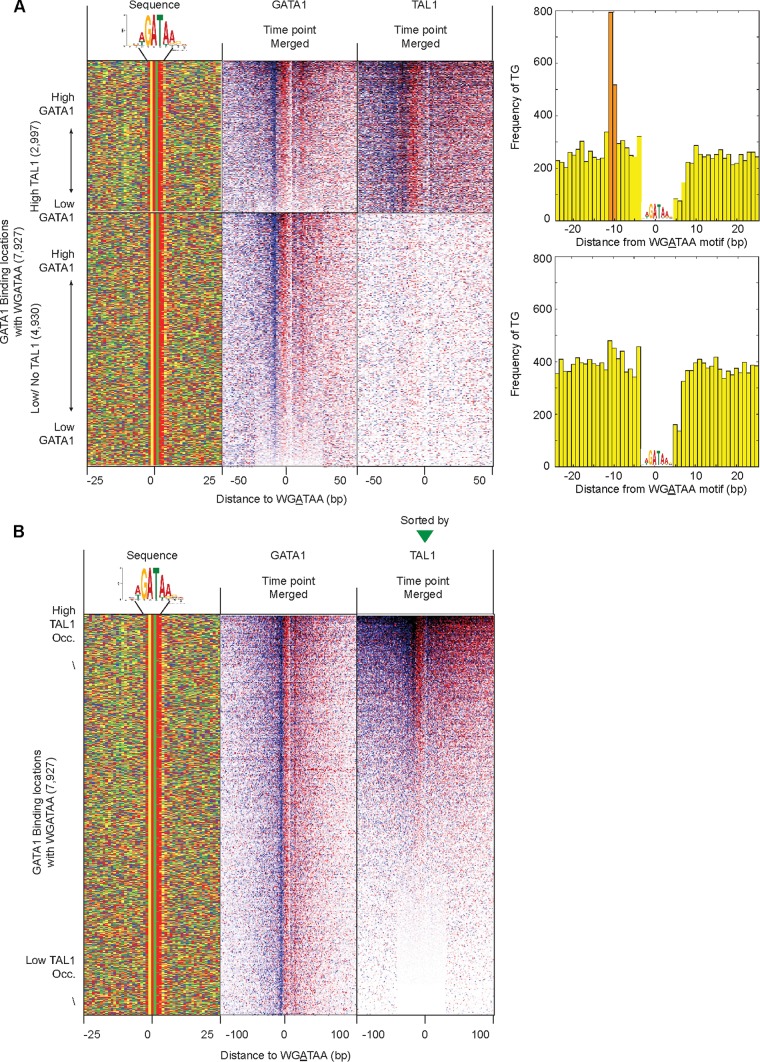
Uniformity of GATA1 and TAL1 cross-linking around WGATAA motifs. (A) Distribution of 5′ ends of GATA1 and TAL1 ChIP-exo sequencing tags around 7,927 WGATAA sites in GATA1 binding locations, comprised of 2,997 GATA1-enriched WGATAA sites (rows) having TAL1 and 4,930 sites lacking TAL1. Both GATA1- and TAL1-cobound WGATAA sites (top) and GATA1-only-bound WGATAA sites (bottom) were sorted by GATA1 occupancy in merged data for all time points. Occupancy was calculated as the sum of total tags around the motif reference point (WGATAA) from 25 bp upstream to 25 bp downstream and 40 bp upstream to 30 bp downstream for GATA1 and TAL1, respectively. (Left) Four-color plot showing the nucleotide compositions of 50-bp surrounding regions of the WGATAA motif: A (red), C (blue), G (gold), and T (green). The bar graphs on the right show the distances of the TG motif from WGATAA sites in GATA1- and TAL1-cobound locations (top) and for GATA1 binding sites with no/low TAL1 occupancy (bottom). (B) TAL1 and GATA1 cross-linking around WGATAA sites sorted by TAL1 occupancy. Shown is the distribution of the 5′ ends of GATA1 and TAL1 ChIP-exo sequencing tags around 7,927 WGATAA sites in GATA1 binding locations. The rows are linked across all the columns and sorted by TAL1 occupancy of merged data for all the time points. TAL1 occupancy was calculated as the sum of tags from 40 bp upstream and 30 bp downstream of the motif reference point (WGATAA). The far-left column shows the nucleotide compositions of regions 25 bp upstream and downstream of the GATA motif reference point.

**FIG 6:**
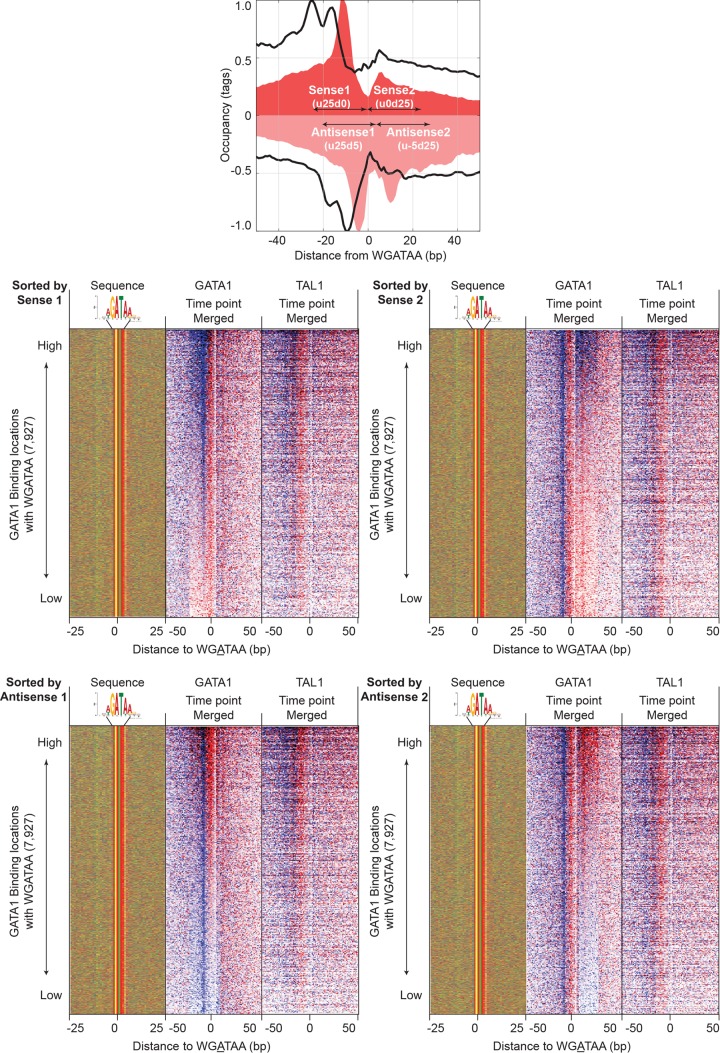
GATA1 and TAL1 tag distribution around WGATAA sites sorted by the occupancy of individual peaks of GATA1 four-peak locations. Tags on the same strand as the motif (blue) and the opposite strand (red) and sequences in the sequence composition plot were sorted by occupancy of sense peak 1 (defined as 0 to 25 bp upstream of the WGATAA reference point) (top left), sense peak 2 (0 to 25 bp downstream) (top right), antisense peak 1 (25 bp upstream and 5 bp downstream) (bottom left), and antisense peak 2 (5 to 25 bp downstream) (bottom right).

The positioning of GATA1 and TAL1 cross-linking points remained consistent relative to WGATAA motifs across most locations (Fig. 5A, sorted by GATA1, and B, sorted by TAL1), including whether GATA1 was associated with TAL1. Hence, the genome-wide average was not a skewed representation of a few sites having high tag counts. Low-occupancy locations often did not contain a full complement of GATA1 tags at each of the four consensus peak locations (i.e., two peak pairs). This was evident when sorting by the signal strength of each of the four peaks separately (Fig. 6). Each peak in a peak pair reflected two distinct measurements of the same cross-link and so should have had roughly equivalent tag counts. The variance in tag counts at each of the four peaks within a location may have multiple sources, including statistical sampling, technical variation in library construction, and double cross-links. The last occurs when a single protein-DNA molecule has two cross-links instead of the more common single cross-link. Since GATA1, on average, has two major points of cross-linking, some fraction of bound molecules may have double cross-links. When this occurs, due to the 5′-3′ directionality of lambda exonuclease, the more 3′ cross-link of a double cross-link will not be observed.

**FIG 7:**
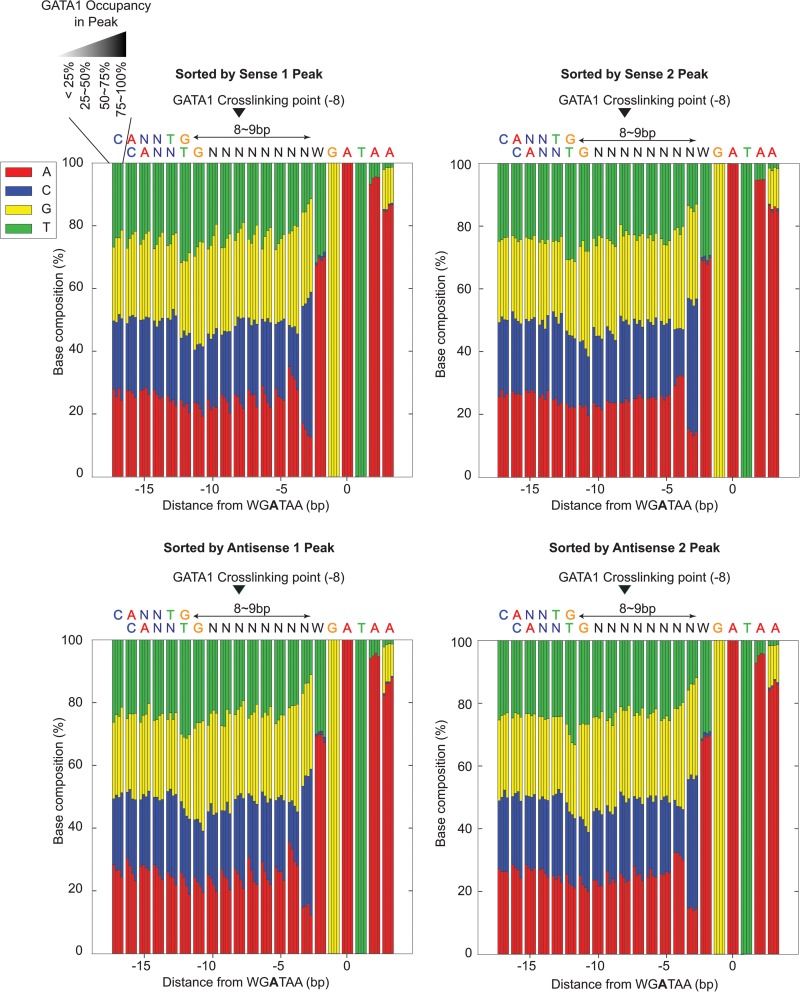
Sequence composition around cross-linking sites when sorted by GATA1 cross-linking levels in individual peaks of 4-peak patterns. Shown is the relationship of nucleotide compositions in bp −18 to +3 from the WGATAA reference point and GATA1 cross-linking levels in individual peaks of GATA1. For each position, the nucleotide compositions in low to high (left to right) cross-linking of an individual peak are shown through 4 adjacent bars.

We also addressed whether potential nucleotide bias in cross-linking efficiency might account for the observed range of GATA1 occupancy. This issue is often raised as a hypothetical concern in ChIP studies. The nucleotide sequence in the vicinity of the major GATA1 cross-linking point (the −8 position from the WGATAA midpoint) deviated little from the overall local average when comparing high versus low GATA1-occupied sites (Fig. 7). The small amount of deviation toward higher G+C frequency at highly occupied sites was also evident in surrounding regions, indicating that it was not specific to the site of cross-linking. Therefore, we conclude that the detection of different binding site occupancy levels is not substantially influenced by putative base-specific differences in cross-linking efficiency.

### TG is enriched 7 or 8 bp upstream of WGATAA at GATA1- and TAL1-cooccupied locations.

We examined the DNA sequence underlying the cooccupancy by GATA1 and TAL1 at all 7,927 identified GATA1-bound WGATAA motifs. Motif locations were first grouped by whether they contained or lacked (i.e., were above or below a set threshold) TAL1 and then sorted by GATA1 occupancy (Fig. 5A). The merged tags of multiple time points (G1E-ER4 cells at 0 h, 3 h, and 24 h for GATA1 and G1E and G1E-ER4 cells at 0 h, 3 h, and 24 h for TAL1) were used to determine occupancy. Where GATA1 and TAL1 cooccupied the same location, their occupancy levels were positively correlated (*R* = 0.42), indicating that binding events are directly or indirectly linked. A TG dinucleotide motif was enriched upstream (more 5′) of the WGATAA motif (Fig. 5A, top, green/yellow vertical stripe) in the GATA1- and TAL1-cooccupied sites, while no TG enrichment was observed in regions having subthreshold levels of TAL1 (Fig. 5A, bottom). The distance between the two closest ends of the two motifs was 7 or 8 bp (i.e., TGN_7–8_WGATAA).

**FIG 8:**
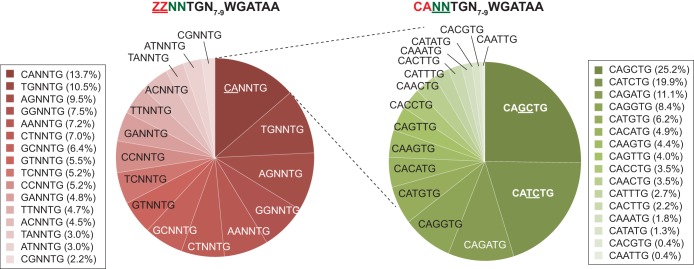
Pie chart of the nucleotide composition of the E-box portion of the composite motif (TG[N_7–8_]WGATAA). When denoting the E-box motif as ZZNNTG, binding locations were first classified by their proportions of ZZ (left). Then, the most frequent CANNTG locations were further classified by their NN contents (right).

One interpretation of this TG dinucleotide is that it comprises the fifth and sixth nucleotides of an E-box (CANNTG) that has been previously linked to WGATAA motifs at TAL1-bound sites *in vivo* (7, 11, 16) and to gene activation (20). We investigated this possibility by inspecting the remaining sequences of a putative E-box configuration, where the conserved TG represented the most WGATAA-proximal side of the E-box. Indeed, matches to an E-box consensus were the most abundant (Fig. 8) but nevertheless represented a minority of all possible configurations. However, ∼50% of all locations contained an A or G (“R” in IUPAC nomenclature) at position 2 of the E-box consensus. Positions 1 and 2 may be somewhat dimorphic in being predominantly MA or BG (M = A/C and B = not C [IUPAC]).

Those with an exact match to the E-box CANNTG motif had the central 2 nucleotides as largely KC dinucleotides (K = G/T). Thus, TAL1-GATA1 genomic binding sites are predominantly (MA/BG)N_2_TGN_7–8_WGATAA, but with a bias toward CAKCTGN_7–8_WGATAA. The tight positional linkage of the (MA/BG)N_2_TG motif with WGATAA and the degeneracy of the remaining sequence in relation to a consensus E-box suggest that DNA interactions at these (MA/BG)N_2_TG motifs contribute to TAL1 binding specificity (in addition to direct or indirect interactions with GATA1 bound at WGATAA).

### Structural relationship between points of TAL1/GATA1 cross-linking and DNA sequence.

We next attempted to orient and position the TAL1/E2A crystal structure (22) to the (MA/BG)N_2_TGN_7–8_WGATAA motif, as illustrated in Fig. 4B. The two primary TAL1 cross-linking points are located 21 and 13 bp upstream of the A at the WGATAA midpoint. Those cross-links flank positions 1 and 2 of NNNNTGn_7–8_WGATAA (positions 1 and 2 are underlined). Making the reasonable assumption that the TAL1 DNA binding alpha helix is what is cross-linking to DNA (22), the most likely placement of the TAL1 alpha helix is midway between its two points of cross-linking and thus within 1 bp of positions 1 and 2. Note that only a single alpha helix of TAL1 is expected to bind DNA, with the other alpha helix in the structure coming from its E2A partner. If this interpretation is correct, then in the context of GATA1, the occurrence of MA or BG dinucleotides at the site of TAL1/DNA contact (positions 1 and 2) supports the crystallographic model in which TAL1 makes fewer overall contacts with DNA than other bHLH proteins (22). In fact, only a single amino acid (E196) makes base-specific contact, which occurs at N-4 of C (in CANNTG). Where A occurs in place of C at position 1, we speculate that N-6 of A might provide the hydrogen bond contact in place of N-4 of C. How BG dinucleotides are accommodated is less obvious.

Given the placement of TAL1 at positions 1 and 2, we suggest its E2A partner contacts the TG dinucleotide (i.e., its complement, CA) at positions 5 and 6 (22, 42) and thus positionally closer to GATA1 along the DNA. This interpretation would place the TAL1/E2A heterodimer in a single predominant orientation (with TAL1 being distal) with respect to GATA1/WGATAA, in addition to being located primarily upstream of WGATAA (i.e., essentially as oriented in the structural model in Fig. 4B).

A second, alternative interpretation of the double peak pairs generated by TAL1 is that one peak pair is from TAL1 and the other is from its partner, in which the heterodimer is directionally oriented relative to the motif (i.e., bound on the 5′ versus the 3′ side of the motif DNA sequence). We deem this to be a more complex and less likely scenario, since cross-linking detection would require that TAL1 cross-link to its partner and that its partner cross-link to DNA. Due to the inherent inefficiency of cross-linking, this is expected to be a low-frequency situation. A third possibility is that TAL1 and its partner can bind in both the forward and reverse orientations. If the heterodimer were binding in both orientations, then in a population of molecules, the cross-linking points should be equidistant and symmetrical from the E-box midpoint. However, that was not observed. Therefore, we favor a model in which TAL1 and its partner have a directional orientation upstream of GATA1 binding, where TAL1 is the more distal partner, and thus, its binding is not being specified directly by the TG motif.

### ChIP-exo versus ChIP-seq in location detection.

**FIG 9:**
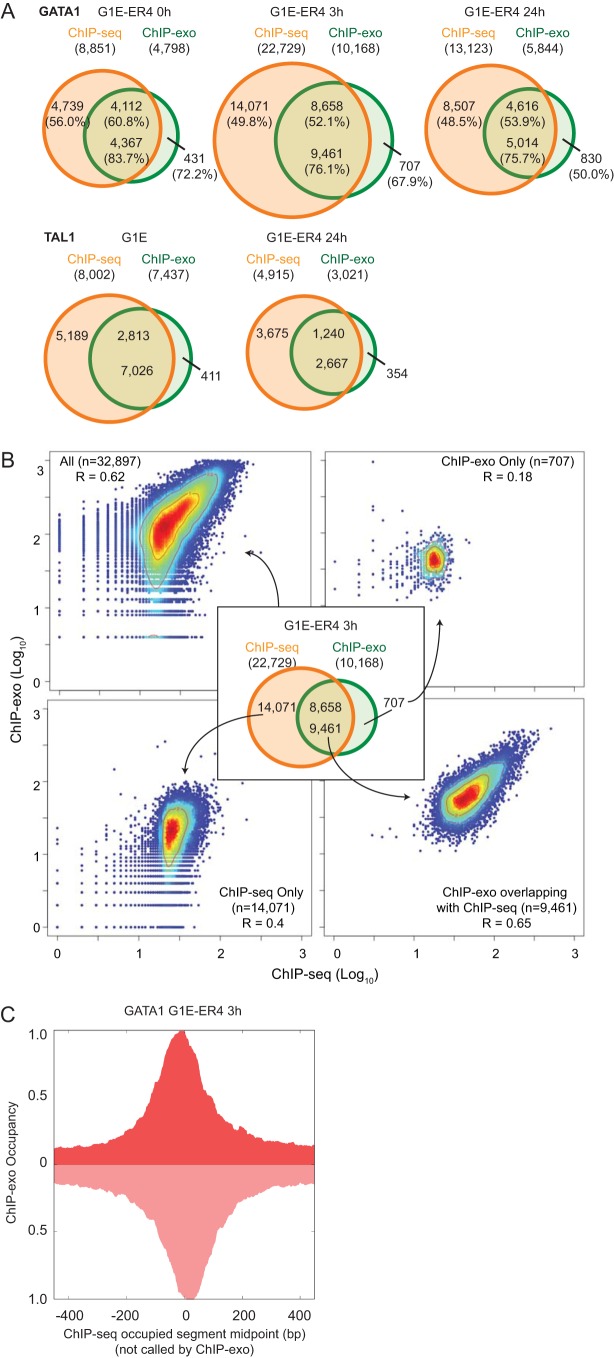
ChIP-exo versus ChIP-seq in location detection. (A) Venn diagram showing overlap of ChIP-exo and ChIP-seq binding locations for GATA1 (G1E-ER4 cells at 0 h, 3 h, and 24 h) and TAL1 (G1E and G1E-ER4 cells at 24 h). The upper numbers correspond to the number of ChIP-seq binding intervals that overlapped with at least one ChIP-exo peak pair location (window size = 80 bp), while the number below is the number of ChIP-exo peak pair locations overlapping with ChIP-seq intervals. The percentages in parentheses are the percentages of locations containing a WGATAA motif <40 bp from the ChIP-exo or ChIP-seq location. (B) Scatter plots with Spearman's correlation coefficient of log_10_-transformed ChIP-exo and ChIP-seq tag counts (window size = 400 bp) around all binding locations of ChIP-exo and ChIP-seq at GATA1 and G1E-ER4 cells at 3 h. The red to blue colors indicate the high to low densities of data points in the scatter plot. Similar results were obtained for the other time point data sets (not shown). (C) Composite distribution of G1E-ER4 3-h cell ChIP-exo reads (smoothing = 20 bp) around occupied segments detected only by ChIP-seq (*n* = 14,071). Similar results were obtained for other time points.

We compared ChIP-exo to a previously existing set of ChIP-seq locations (15) performed in the same cell system, with the intention of understanding why some locations were called by one method and not by the other. For ChIP-exo, we used the set of locations defined by the intersection of peak pairing and MultiGPS (filtered to be >2-fold over background; *q* value < 0.05). This high stringency gives stronger confidence in the peak calls, but at the cost of some false negatives. The sets of ChIP-exo and ChIP-seq locations showed substantial overlap (Fig. 9A), as expected, since ChIP-exo is essentially a refined version of ChIP-seq. A high percentage (50 to 72%) of ChIP-exo locations that did not overlap contained WGATAA motifs (motif *P* value < 10^−3^) <40 bp away, some of which are likely to be false negatives. This compares with 75 to 84% of ChIP-seq/ChIP-exo intersects having a motif. About half of all ChIP-seq locations that did not overlap ChIP-exo were deemed to be enriched with false negatives by the same criteria. Taken at face value, the nonoverlapping locations (outersects) may be binding events detectable in only one assay. Alternatively, thresholding of the data may result in a set of locations being marginally above a threshold in one assay and marginally below a threshold in the other assay.

We tested this by directly comparing occupancy levels between the two assays. A scatter plot comparison of occupancy levels from ChIP-seq versus ChIP-exo showed that they were well correlated (Spearman *R*, ∼0.65) (Fig. 9B) when both assays identified GATA1 locations. The correlation dropped to 0.4 when locations were identified only by ChIP-seq. Locations identified by only one assay typically had low occupancy in both assays. Therefore, the lack of a call in the ChIP-exo assay is more likely due to differences in stringency thresholding between the two assays rather than a qualitative distinction between the ChIP-seq and ChIP-exo assays.

To further address the ChIP-exo/ChIP-seq correspondence of the outersects, we examined the ChIP-exo GATA1 tag distributions around the midpoints of occupied segments bound only by ChIP-seq. On average, the ChIP-exo peak pairs were centered on the ChIP-seq-only midpoint locations (Fig. 9C), demonstrating that they were indeed reporting on similar locations despite being below the detection threshold.

Taken together, many of the locations called in only one of the two assays likely reflect a substantial amount of real but low-occupancy binding events. While such events can be captured, they will also have a higher false-positive rate. The primary difference between called locations in the two assays lies in the level of false discovery (the sum total of false positives and negatives), defined by assay-specific location-calling thresholds. While the use of thresholds adds confidence to location calling by reducing false positives, it comes at the price of creating false negatives.

### Dynamics of GATA1 and TAL1 binding during erythroid development.

**FIG 10:**
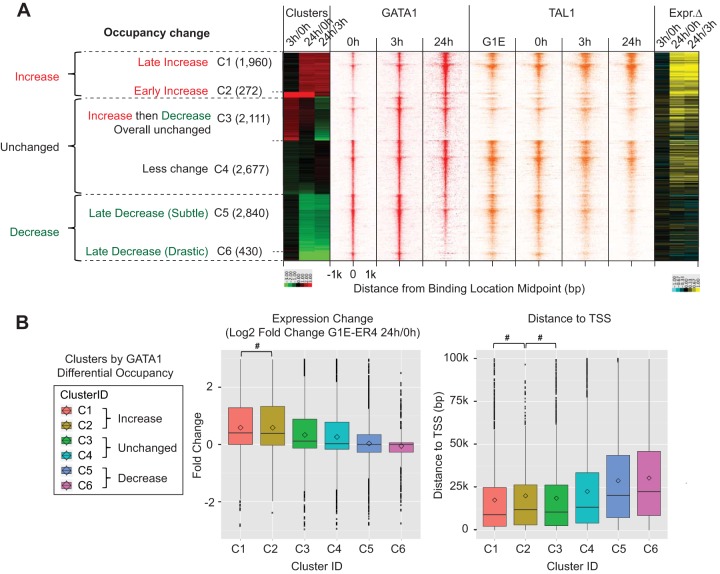
Dynamics of GATA1 and TAL1 binding during erythroid development. (A) (Left) Heat map of GATA1 and TAL1 total-tag-count-normalized tags (strand merged) plotted around GATA1 binding locations, which were clustered by differential occupancy of GATA1 between time points. Differential occupancies between time points (G1E-ER4 cells at 3 h/0 h, 24 h/0 h, and 24 h/3 h) were determined as the log_2_-fold change in the ratio of GATA1 tags over the input control between time points using EdgeR and MultiGPS. (Right) For each binding location, a heat map of log_2_-fold expression change (Expr. Δ) of the closest gene between G1E-ER4 cells at 3 h/0 h, 24 h/0 h, and 24 h/3 h is shown, where increased, unchanged, and decreased expression are depicted as yellow, black, and blue, respectively. (B) Box plot of expression change (G1E-ER4 cells at 24 h/0 h) (left) and distance to the closest transcription start site (right). Differences that were insignificant (*P* > 0.05) after examining the differences among pairwise combinations of all clusters, using a Mann-Whitney test, are indicated (#).

The erythroid developmental program has already been well described based on ChIP-seq. Since the ChIP-exo data we obtained substantially overlapped the ChIP-seq locations, we fully expect that linked genes would also be involved in various erythroid pathways. To confirm this, we examined the changes in GATA1 binding 0, 3, and 24 h after GATA1 activation. The set of ∼10,000 GATA1 binding locations were classified by their differential occupancies between various induced differentiation time points (3 versus 0 h, 24 versus 0 h, and 24 versus 3 h) (Fig. 10A). Six kinetic classes were produced by *k*-means clustering, which were grouped into increased (clusters 1 and 2), unchanged (clusters 3 and 4), and decreased (clusters 5 and 6) GATA1 occupancy. Since GATA1 was ectopically induced, we assume that its total level of genome-wide binding would either increase or stay approximately the same. We normalized the total of all occupancy levels at each time point to be constant across the time points. Therefore, an apparent relative “decrease” in GATA1 occupancy upon GATA1 induction may actually reflect less of an increase than other locations. In general, the kinetic patterns of TAL1 occupancy were similar to those of GATA1, as expected when binding as a complex (Fig. 10A, right). We note that prior to induction (0 h), GATA1 is in both the cytoplasm and the nucleus (42) and thus has a significant level of binding genome-wide. This preactivation state is insufficient to promote differentiation, and it may be inactive until released by estradiol.

The closest annotated mouse gene was assigned to each GATA1 binding location to examine the effects of GATA1 and TAL1 binding on gene expression. These locations were largely associated with genes involved in blood cell development and maintenance, as previously determined (references 35 and 42 and data not shown). The binding locations with the greatest increase in GATA1 occupancy displayed the greatest increase in gene expression of the nearest gene in response to GATA1 activation (15) (Fig. 10B, clusters 1 and 2). GATA1 was closer to their TSS on average than the average of all other genes linked to a GATA1 location that did not experience the same relative increase in GATA1 occupancy (i.e., clusters 3 to 6). Therefore, where GATA1 occupancy differentially increases near a TSS, its gene may be activated during erythroid differentiation.

## DISCUSSION

In this study, we determined the genome-wide positional organization of GATA1 and TAL1 at nearly single-base-pair resolution using ChIP-exo. Comparison of location calling using peak pairing versus MultiGPS revealed that they largely call the same locations. Peak pairing picks up low-complexity binding locations that are missed by MultiGPS, whereas MultiGPS picks up locations where tags are missing on one strand (likely due to molecule-specific biases arising during sample preparation and library construction). These are missed by peak pairing. Often the biggest differences in called locations are due to differences in data thresholding (tag counts and patterning). A large fraction of locations typically fall near the threshold, and thus, small differences in thresholding can create an appearance of incongruence between location-calling methods. We similarly compared ChIP-exo to ChIP-seq and found them to be highly similar, with the main differences attributable to differences in false-discovery rates and thresholding.

We identified about 10,000 GATA1-bound WGATAA sites in mouse G1E cells containing ectopically expressed GATA1 and about 15,000 TAL1 locations. About 3,000 of these locations correspond to TAL1/GATA1 complexes bound to a WGATAA motif having a TG dinucleotide enriched 7 or 8 bp upstream. TAL1, and presumably its E2A partner, bind stereospecifically to GATA1-bound DNA. Our assessment of the cross-linking pattern leads us to propose that TAL1 contacts DNA more distally upstream of WGATAA than E2A. We also found evidence of TAL1 being in close proximity to DNA on the downstream side of GATA1, but the structural basis for this is unknown.

The structural model proposed here gives a basis for mechanistic hypotheses about the many functions of TAL1 and GATA1. TAL1 plays essential roles at multiple stages of hematopoiesis, including specification of hematopoietic cell lineages and prevention of ectopic cardiogenesis in the multipotent cardiovascular endoderm (43), establishment of early hematopoietic stem and progenitor cells (44–46), and differentiation to produce erythroid cells and several myeloid cells (47). These pleiotropic effects are accomplished by dynamic changes in the genome-wide binding profiles of TAL1 during differentiation, leading to TAL1 occupancy at regulatory regions distinctive of each cell type (7, 24, 33, 48–51). These large-scale changes in TAL1 occupancy during differentiation appear to be driven, at least in part, by TAL1 binding, together with a GATA factor, which provides a major component of the sequence specificity (7, 24, 51). Our structural model for sites of cooccupancy shows sequence-specific interaction of GATA1 zinc fingers with the binding site motif, WGATAA, and the E2A heterodimeric partner of TAL1 binding to a partial E-box. However, the interaction of TAL1 with DNA does not show detectable sequence specificity. By assuming a similar structure for TAL1 cobound with other GATA factors (e.g., GATA2 in hematopoietic stem and progenitor cells and GATA4 in multipotent endoderm), one can envision a mechanism whereby TAL1 binds specifically to a distinct set of regulatory regions in different cell types. The model suggests that TAL1 is not guided primarily by binding its preferred binding site in solution (an E-box) but rather is guided by its interaction with a GATA factor that is strongly bound to its DNA binding site motif. Thus, the cell-type-specific binding by a series of paralogous GATA factors could be a major determinant of the cell-specific binding profiles for GATA1. These sites of cooccupancy by GATA factors and TAL1 are strongly associated with gene induction (12, 50, 51), a result that is recapitulated by our high-resolution mapping here. Multiple mechanisms of GATA1-dependent repression have been proposed, each applying to a different subset of genes (8, 10). During the specification of hematopoietic versus cardiac cell fates in mesodermal cells, TAL1 not only activated genes needed for hematopoiesis, but prevented the retention of active chromatin marks at enhancers needed for the cardiac lineage (51). Our structural model suggests possible explanations for the different effects of TAL1. In the presence of a GATA factor, a structure with TAL1-E2A bound upstream from GATA may serve as a platform for recruitment of activators and coactivators in induction. Once the GATA factor is no longer present, the remaining TAL1 may assume a different position or conformation that interferes with the recruitment of positive regulators, leading to loss of induction.

The high resolution of ChIP-exo allowed us to identify DNA segments bound by multiple molecules of GATA1 or TAL1 in a cluster. These clusters were not resolved by ChIP-seq. The clusters of homotypic binding represent a small but significant subset of all the bound locations. The significance of this is unclear, although it might reflect constraints imposed by higher-order structures that involve multiple copies of GATA1 and TAL1. Our analyses did not identify major functional distinctions between clustered and nonclustered locations, apart from the former being more highly expressed on average. Nonetheless, G1E differentiation along the hematopoietic lineage upon ectopic activation of GATA1 is linked to a substantial increase in GATA1/TAL1 binding to about 3,000 sites genome-wide.

## Supplementary Material

Supplemental material
