# Supplementary material for: Genome-Wide Organization of GATA1 and TAL1 Determined at High Resolution
Source: Mol Cell Biol. 2015 Dec 18;36(1):157–72. doi: 10.1128/MCB.00806-15 (PMC4702602; doi:10.1128/MCB.00806-15)
Supplement: Supplemental material [file supp_36_1_157__index.html]

Supplemental material 

# Genome-wide organization of GATA1 and TAL1 determined at high resolution

## Supplemental material

- Supplemental file 1 -

  Table S1 (Sequencing read counts)

  XLSX, 50K
- Supplemental file 2 -

  Table S2 (Final GATA1 binding locations)

  XLSX, 1.4M
